# Supplementary material for: Mechanisms governing the pioneering and redistribution capabilities of the non-classical pioneer PU.1
Source: Nat Commun. 2020 Jan 21;11:402. doi: 10.1038/s41467-019-13960-2 (PMC6972792; doi:10.1038/s41467-019-13960-2)
Supplement: Supplementary file 7 — Source data [file 41467_2019_13960_MOESM7_ESM.zip › Source_Data/Figure6/Figure6B_MotifScanOutput/FLI1_mutPU.1/homerResults/motif6.similar.html]

motif6

## Information for motif6

A
C
G
T
A
T
G
C
A
C
G
T
A
C
G
T
C
G
T
A
A
C
G
T
A
G
T
C
C
G
A
T
T
A
G
C
G
C
A
T
  
Reverse Opposite:  

C
G
T
A
A
T
C
G
C
G
T
A
A
C
T
G
C
G
T
A
A
C
G
T
C
G
T
A
C
G
T
A
T
A
C
G
T
G
C
A
  

|  |  |
| --- | --- |
| p-value: | 1e-190 |
| log p-value: | -4.387e+02 |
| Information Content per bp: | 1.716 |
| Number of Target Sequences with motif | 1820.0 |
| Percentage of Target Sequences with motif | 17.74% |
| Number of Background Sequences with motif | 2824.1 |
| Percentage of Background Sequences with motif | 7.41% |
| Average Position of motif in Targets | 99.8 +/- 53.7bp |
| Average Position of motif in Background | 99.3 +/- 63.2bp |
| Strand Bias (log2 ratio + to - strand density) | 0.0 |
| Multiplicity (# of sites on avg that occur together) | 1.13 |
| Motif File: | file (matrix) reverse opposite |

### Similar de novo motifs found

|  |  |  |  |  |  |  |  |
| --- | --- | --- | --- | --- | --- | --- | --- |
| Rank | Match Score | Redundant Motif | P-value | log P-value | % of Targets | % of Background | Motif file |
| 1 | 0.978 | A G T C C G T A A T G C C G T A A C T G C G T A A G C T C G T A C G T A T C A G T C G A | 1e-184 | -425.188714 | 17.99% | 7.71% | motif file (matrix) |
| 2 | 0.969 | C G T A A C T G C G T A A C G T C G T A C G T A T A C G | 1e-176 | -406.207511 | 19.64% | 9.05% | motif file (matrix) |
| 3 | 0.956 | G A C T A G C T A G T C C G A T A C G T C G T A C G A T T A G C C G A T | 1e-176 | -405.470165 | 16.94% | 7.18% | motif file (matrix) |
| 4 | 0.943 | A T G C A G C T C G A T C G T A C G A T A G T C C G A T A T C G | 1e-174 | -401.218040 | 15.93% | 6.54% | motif file (matrix) |
| 5 | 0.977 | A G C T A G T C A G C T A C G T C G T A A C G T A G T C C G A T A T C G G A C T C A G T G A C T | 1e-170 | -391.827745 | 14.64% | 5.78% | motif file (matrix) |
| 6 | 0.922 | C G T A A C T G C G T A A C G T C T G A C T G A G T A C T C A G | 1e-90 | -207.833181 | 14.05% | 7.35% | motif file (matrix) |
| 7 | 0.868 | T A C G C G T A A C T G T C G A G A C T C G T A C T G A T C A G G T C A G T C A C G A T T A G C G C T A | 1e-66 | -152.215462 | 4.41% | 1.42% | motif file (matrix) |
